# Melatonin alleviates titanium nanoparticles induced osteolysis via activation of butyrate/GPR109A signaling pathway

**DOI:** 10.1186/s12951-021-00915-3

**Published:** 2021-06-06

**Authors:** Yanglin Wu, Fan He, Chenhui Zhang, Qin Zhang, Xinlin Su, Xu Zhu, Ang Liu, Weidong Shi, Weifeng Lin, Zhongqin Jin, Huilin Yang, Jun Lin

**Affiliations:** 1 Department of Orthopaedics, The First Affiliated Hospital of Soochow University, Soochow University, No. 188 Shizi Street, Suzhou, 215006 Jiangsu China; 2 Orthopaedic Institute, Medical College, Soochow University, Suzhou, 215007 China; 3 Department of Digestive, Children’s Hospital Affiliated to Soochow University, Suzhou, China; 4 Department of Materials and Interfaces, Weizmann Institute of Science, 76100 Rehovot, Israel

**Keywords:** Gut microbiota, Inflammatory osteolysis, NLRP3 inflammasome, Butyrate, GPR109A

## Abstract

**Background:**

Inflammatory osteolysis after total joint replacement (TJR) may cause implant failure, periprosthetic fractures, and be a severe threat to global public health. Our previous studies demonstrated that melatonin had a therapeutic effect on wear-particles induced osteolysis. Gut microbiota is closely related to bone homeostasis, and has been proven to be affected by melatonin. However, whether melatonin could play its anti-osteolysis effects through reprogramming gut microbiota remains elusive.

**Results:**

Here, we demonstrated that melatonin could alleviate Ti-particles induced osteolysis, while this therapeutic effect was blocked by antibiotic cocktail treatment. Interestingly, transplantation of fecal microbiota from mice treated with melatonin reappeared the same beneficial effect. Analysis of the 16S rRNA revealed that melatonin could reverse dysbacteriosis triggered by osteolysis, and elevate the relative abundance of some short chain fatty acid (SCFA) producing bacteria. Moreover, butyrate was enriched by exogenous melatonin administration, while acetate and propionate did not show an evident difference. This was consistent with the results of the metagenomic approach (PICRUSt2) analysis, which revealed a general increase in the synthetic enzymes of butyrate. More importantly, direct supplementation of butyrate could also recapitulate the anti-osteolysis effect of melatonin. Further analysis identified that butyrate alleviated osteolysis via activating its receptor GPR109A, and thus to suppress the activation of NLRP3 inflammasome triggered by Ti-particles.

**Conclusions:**

Taken together, our results suggested that the benefits of melatonin mainly depend on the ability of modulating gut microbiota and regulating butyrate production.

**Graphic Abstract:**

**Figure:**
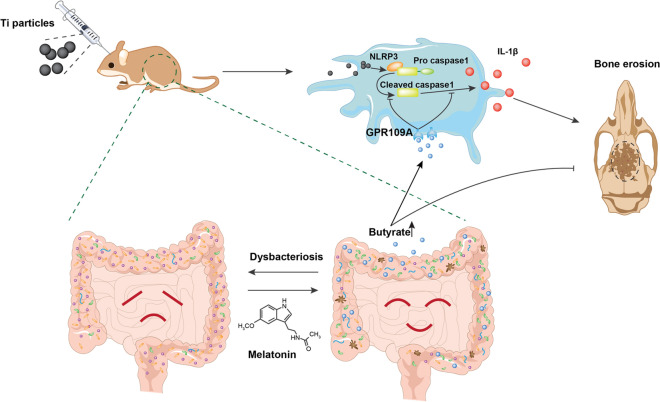

**Supplementary Information:**

The online version contains supplementary material available at 10.1186/s12951-021-00915-3.

## Background

As the essential treatment for end-stage arthritis, total joint replacement (TJR) could effectively relieve joint pain, stiffness, and improve joint function [1]. However, periprosthetic aseptic osteolysis caused by wear particles is still the major long-term complication after total joint replacement. Unfortunately, it is reported that the incidence rate is generally between 5 and 20%, and it can be even as high as 40% in some studies [2]. Periprosthetic osteolysis may result in periprosthetic fractures, implant loosening, and complex revision surgery. It is not only a heavy economic burden, but also brings an increased risk of complications.

Recent evidence demonstrates that osteolysis induced by prosthetic-wear particles have a closely correlation with NLRP3 inflammasome activation [3–6]. The NLRP3 inflammasome is well-characterized by its ability of controlling the activation of caspase-1 under pathological stimulations [7–9]. It is mainly composed of sensor protein, proinflammatory caspase-1, and adaptor protein apoptosis related spot like protein [10–12]. As the vital innate immune sensor, the NLRP3 inflammasome could be stimulated by DAMPs (diverse damage-associated molecular patterns) such as excess glucose [13], ATP [7], urate [14], and cholesterol crystals [15]. Several studies have determined that prosthetic-wear particles induced osteolysis had an evident connection with NLRP3 inflammasome activation [3–6]. More recently, Jämsen et al*.* [16] confirmed that the NLRP3 inflammasome stimulation was responsible for wear particles inducing immune response in human macrophages. Therefore, any method that can suppress the activation of NLRP3 inflammasome will become the potential treatment for periprosthetic osteolysis.

Melatonin, as a rhythm controlling hormone secreted by the pineal gland, is associated with extensive physiological functions, including circadian rhythms, anti-tumor, anti-inflammatory, and antioxidant [17–20]. Numerous of previous studies have reported direct benefits of melatonin on these physiological disorders. However, only a few studies have focused on its indirect benefits mediated through gut microbiota reprogramming. Yin et al*.* [21] firstly reported that melatonin administration could reverse the dysbacteriosis and elevate the concentration of acetate, which improved lipid dysmetabolism in mice with a high fat diet. As major products of the microbial fermentation, SCFAs are mainly composed of acetate, propionate, and butyrate [22]. SCFAs can be metabolized from dietary fibers, proteins, and peptides via gut microbiota [23]. Recent studies suggested that butyrate, an important component of SCFAs, can exert anti-atherosclerotic action via suppressing the activation of NLRP3 inflammasome in endothelial cells [24].

Although several studies have revealed the benefits of melatonin on osteolysis [25–28], whether melatonin plays the anti-osteolysis effects indirectly through gut microbiota reprogramming is still unknown. The role of SCFAs in the course of osteolysis is also undiscovered. Therefore, in this study, we investigated whether melatonin could reprogram the gut microbiota in a normal diet and increase the concentration of SCFAs. Then, we elucidated why melatonin administration only raised the concentration of butyrate. Soon afterwards, we tested the hypothesis that butyrate could attenuate Ti-particles induced osteolysis via inhibiting the activation of NLRP3 inflammasome. Finally, we also confirmed that the beneficial effect of butyrate was dependent on its receptor GPR109A.

## Results

### Gut microbiota is associated with the benefits of melatonin in preventing osteolysis

**Fig. 1:**
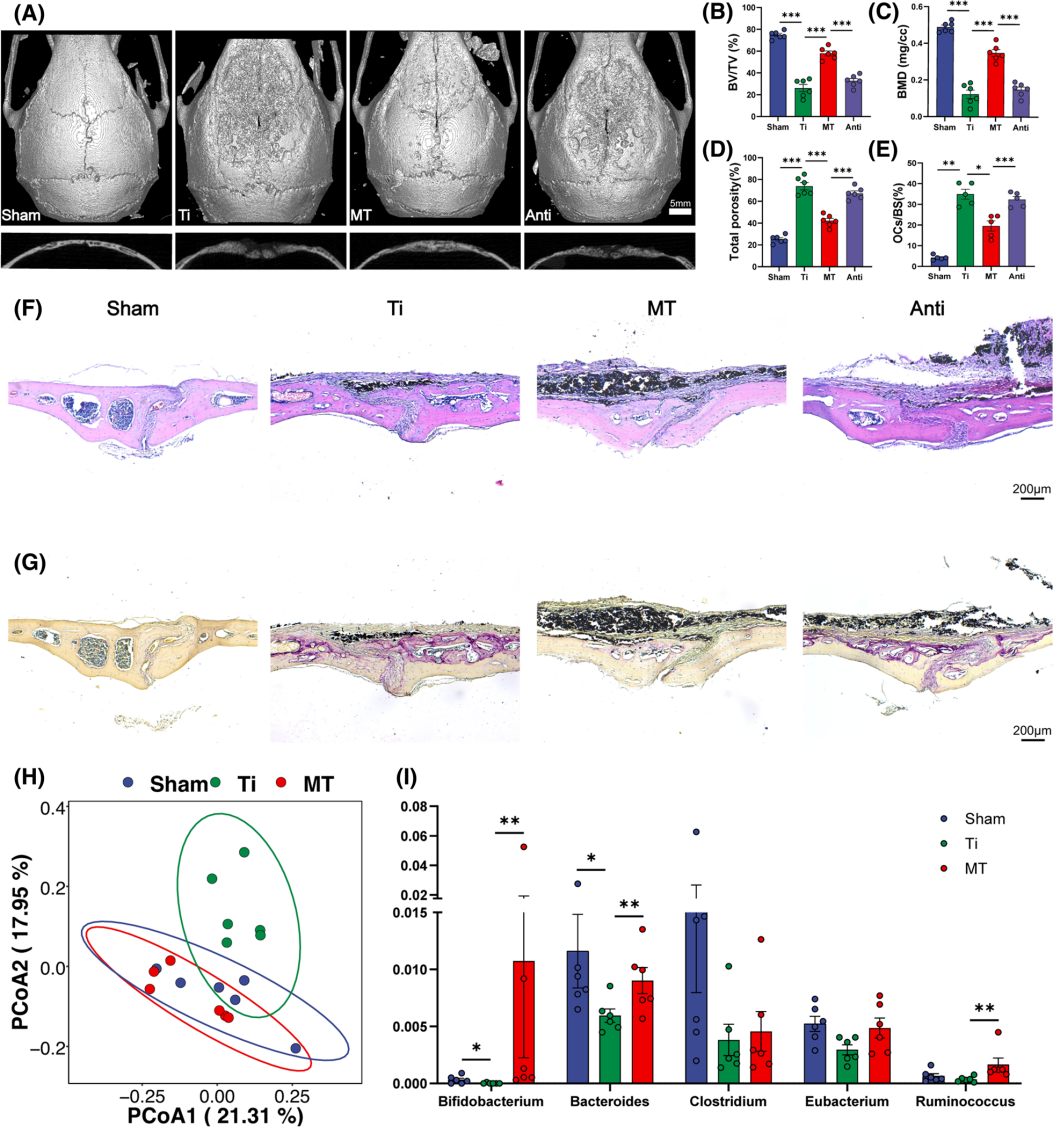
The benefits of melatonin was correlated with gut microbiota. **A** Representative view of calvarium in each group via Micro-CT 3D reconstruction. **B**–**D** Quantification of bone erosion parameters (BV/TV) bone volume to tissue volume ratio, (BMD) bone mineral density, and total porosity. n = 6. **E** Percentage of osteoclasts surface per bone surface (OCs/BS, %). n = 5. **F** H&E and **G** TRAP staining of calvarium slices from each group. **H** Principle coordinate analysis (PCoA) plot from sham (PBS), Ti (Ti exposure), and MT (Ti exposure and melatonin treatment) groups based on the Bray Curits distance. n = 6. **I** Relative abundance of SCFA producing microbes in mice feces. n = 6. Results are expressed as mean ± SEM (One-way ANOVA[post hoc:SNK] *p < 0.05, **P < 0.01, ***p < 0.001)

Although our previous studies have demonstrated the benefits of melatonin on inflammatory osteolysis [25, 26], whether melatonin plays the anti-osteolysis effects indirectly through gut microbiota reprogramming is still unknown. Gut microbiota is well known to be affected by antibiotics [32–35]. Antibiotic cocktail treatment could greatly reduce gut bacterial amount [36, 37]. To determine whether the anti-osteolysis effect of melatonin was mediated by gut microbiota, we treated Ti-particles exposed mice with melatonin and antibiotic cocktail simultaneously. As expected, the therapeutic effect of melatonin was nearly blocked by antibiotic cocktail treatment (Fig. 1A). Bone erosion was significantly aggravated in antibiotic cocktail and melatonin treated mice (Anti group) with a lower level of BV/TV, BMD, a higher level of total porosity, and a higher ratio of OCs/BS in comparison with that in melatonin treated mice (Fig. 1B–E). HE and TRAP staining also confirmed that antibiotic cocktail treatment could diminished the benefits of melatonin with more erosion area and osteoclasts activation (Fig. 1F, G). These results suggested that gut microbiota was closely related to the benefits of melatonin on osteolysis.

### Melatonin reverses the disorder of gut microbiota and enriches the SCFA-producing bacteria abundance

After confirming that the benefits of melatonin were associated with gut microbiota, we further determined the overall structural changes of gut microbiota in response to melatonin administration by analysis of the 16S rRNA gene sequences of microbial samples collected from feces of mice in Sham, Ti, MT groups. Bray Curits distance-based PCoA (principal coordinate analysis) showed different clustering of gut microbiota community between the Ti and Sham group after two weeks of intervention (Fig. 1H). At the meantime, the mice in the MT and Ti group also had different composition of gut microbiota, while Sham and MT group shared a similar composition in gut microbiota, indicating that melatonin administration may reverse the dysbacteriosis triggered by Ti-particles induced osteolysis.

In the view of this phenomenon, we further applied Linear discriminant analysis Effect Size (LEfSe) analysis (Additional file 1: Figure S1), which is a common method for effect size estimation, tests of biological consistency, and class comparison [38]. Compared with Ti group, the genera of *Bifidobacterium, Bacteroides and Ruminococcus* were significantly enriched in the MT group (Fig. 1I). Interestingly, these enriched genera were positively associated with the production of SCFAs, which were metabolized from dietary fibers by the gut microbiota [39]. In general, melatonin administration could enrich SCFAs-producing bacteria in the Ti-particles-induced osteolysis model, which may be closely related to the protective effect of melatonin against osteolysis.

### Melatonin enriches the relative abundance of butyrate related enzyme and raises butyrate concentration

**Fig. 2:**
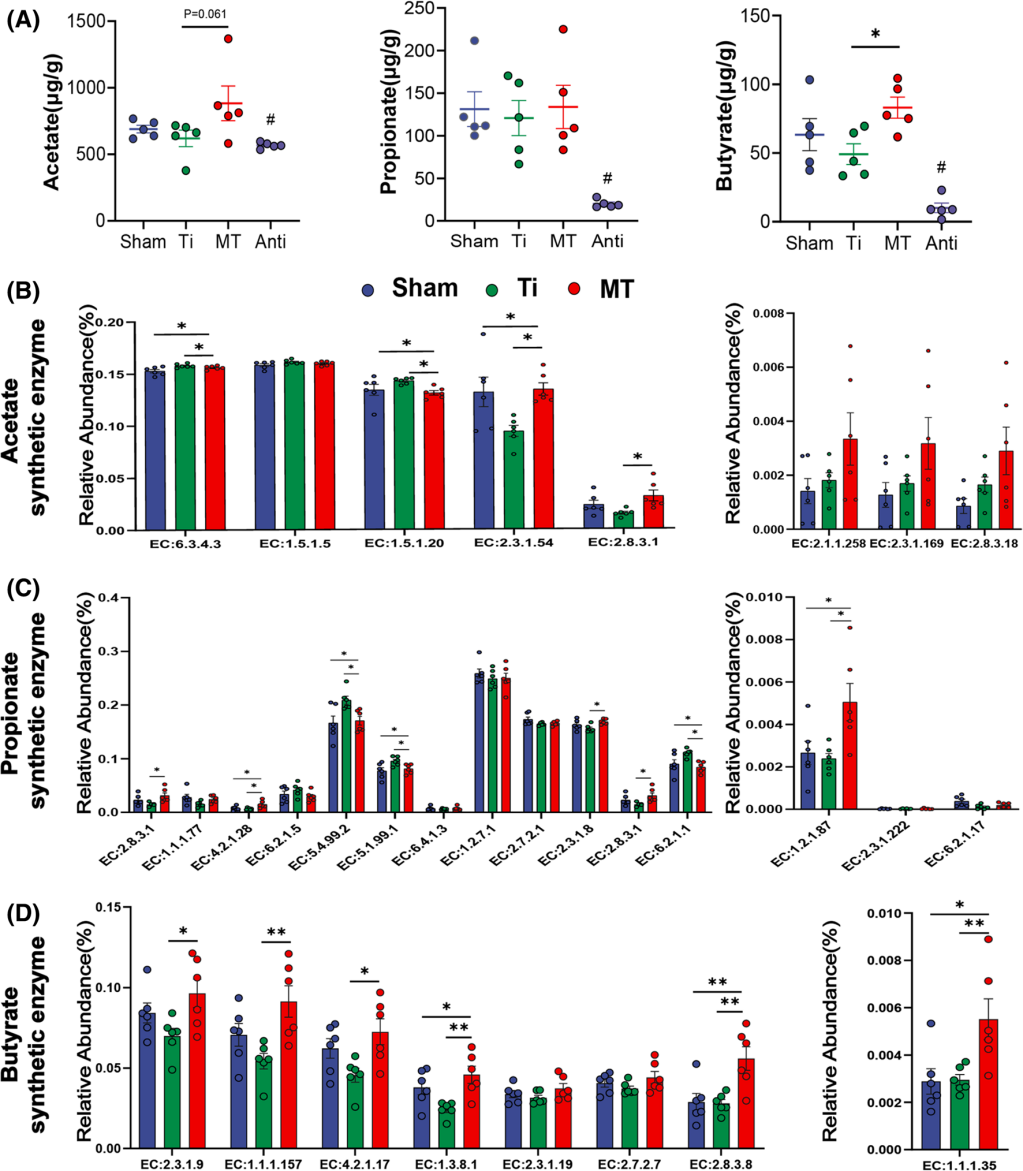
Melatonin enriched the abundance of butyrate related enzymes and fecal butyrate concentration in mice. **A** The concentration of acetate, propionate, and butyrate in fecal samples. n = 6. **B**–**D** Relative abundance of acetate, propionate, and Butyrate synthesis related enzymes from PICRUSt2. n = 6. Results are expressed as mean ± SEM (One-way ANOVA[post hoc:SNK] *p < 0.05, **P < 0.01, ***p < 0.001, # significantly different from Sham, Ti and MT group)

To determine the effect of changes in SCFA-producing bacteria on short chain fatty acids, we measured the SCFAs concentration of the feces collected from mice in each group through targeted metabolomics analysis. The result revealed that the concentration of butyrate was dropped a little bit after osteolysis surgery and melatonin administration could increase it nearly to twofold level of Ti group, while the concentration of propionate did not show significantly change and acetate was raised a little (P = 0.061) between Ti and MT group (Fig. 2A). Encountered with this interesting phenomenon, we analyzed the metabolic function profile using a metagenomic approach (PICRUSt2). PICRUSt2 is a new approach of functional genes prediction for 16S rRNA, which has been proven to be more accurate than any other approaches overall in metagenome inference [40]. Amazingly, most of butyrate synthesis related enzymes identified in KEGG and Metacyc databases were significantly enriched by melatonin supplementation (Fig. 2D). However, we did not observe a similar trend in acetate and propionate. The expression of some metabolic enzymes, such as EC:6.3.4.3, EC:1.5.1.20 (acetate) and EC:5.4.99.2, EC:5.1.99.1, EC6.2.1.1 (propionate), were obviously higher in Ti group (Fig. 2B, C). Collectively, these results demonstrated that melatonin could elevate the fecal concentration of butyrate via enriching the abundance of enzymes required for butyrate synthesis, which may be essential for its anti-osteolysis ability.

### Fecal microbiota transplantation from melatonin treated mice has the same protective effect against osteolysis

**Fig. 3:**
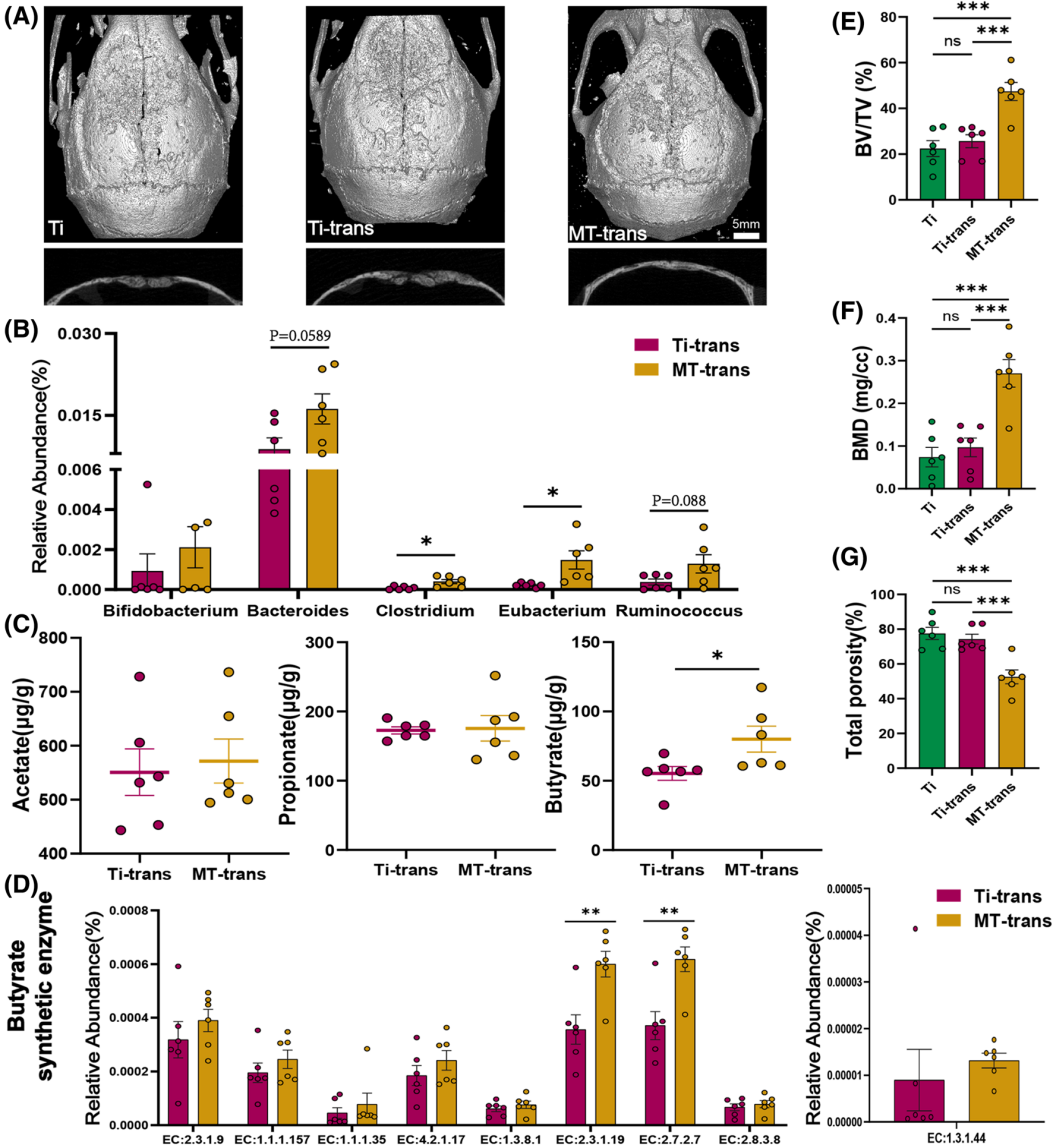
The anti-osteolysis effect of melatonin could be transmitted by fecal microbiota transplantation. **A** Representative view of calvarium in each group via Micro-CT 3D reconstruction. **B** Relative abundance of SCFA producing microbes in fecal samples n = 6. **C** Three major SCFA concentration in fecal samples. n = 6. **D** Butyrate synthesis related enzymes. n = 6 in each group. Results are expressed as mean ± SEM (Unpaired t-tests * p < 0.05, ** p < 0.01). **E**–**G** Quantification of bone erosion parameters (BV/TV) bone volume to tissue volume ratio, (BMD) bone mineral density, and total porosity. n = 6. Results are expressed as mean ± SEM (One-way ANOVA[post hoc:SNK] ***p < 0.001)

Based on previous reports [36, 37, 41], antibiotic cocktail treatment could greatly reduce the amount of gut bacteria, and the microbial amount could substantially rise after microbiota transplantation. To confirm the benefits of melatonin mediated by gut microbiota, mice were transplanted with microbiota from Ti or MT group after antibiotic cocktail treatment. As hypothesized, mice received microbiota from MT group revealed the attenuated course of osteolysis, similar to their donors. The result of Micro-CT analysis (Fig. 3A, E–G), H&E and TRAP staining (Additional file 1: Figure S2A, B) revealed mice in MT-trans group (fecal microbiota transplantation from mice in MT group) had a similar anti-osteolysis therapeutic effect. Both TRAP positive cell number and OCs/BS were almost reduced by 50% (Additional file 1: Figure S2E, F). However, these protective effects were not observed in Ti-trans (fecal microbiota transplantation from mice in Ti group) group. These results suggested that the benefits of melatonin could be transmitted by fecal microbiota transplantation, which further confirmed that the gut microbiota was indispensable in the beneficial actions of melatonin. Meanwhile, Bray Curits distance-based principal coordinate analysis (PCoA) revealed that MT-trans and Ti-trans groups shared different structure of gut microbiota (Additional file 1: Fig S3). More importantly, there was still an increasing trend in the genera of SCFAs-producing bacteria, such as *Bifidobacterium, Bacteroides, Clostridium, Eubacterium, Ruminococcus* (Fig. 3B). Similarly, the concentration of butyrate in MT-tans group was elevated after fecal microbiota transplantation, while acetate and propionate showed no tendency (Fig. 3C). This result was consistent with PICRUSt2 analysis. Butyrate synthesis related enzymes were generally increased, while the other two main short chain fatty acids synthetic enzymes were not evidently affected (Fig. 3D, Additional file 1: Figure S2C, D). Taken together, the anti-osteolysis effects of melatonin could be transmitted by fecal microbiota, and microbial metabolite butyrate may be responsible for the indirect benefits of melatonin.

### Butyrate alleviates the osteolysis via inhibiting NLRP3 inflammasome activation

**Fig. 4:**
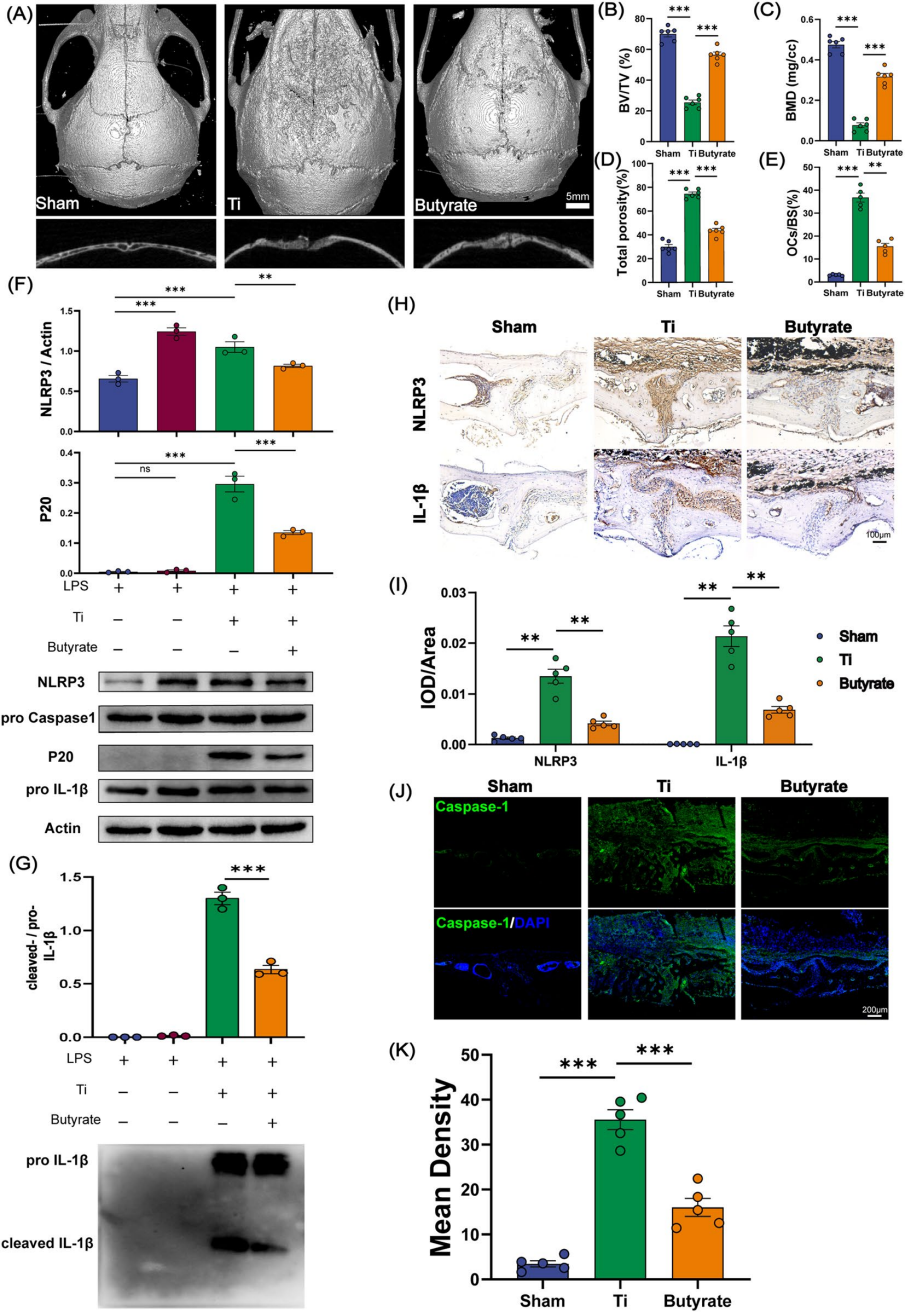
Butyrate suppressed NLRP3 inflammasome activation in vivo and in vitro. **A** Representative view of calvarium in each group via Micro-CT 3D reconstruction. **B**–**D** Quantification of bone erosion parameters (BV/TV) bone volume to tissue volume ratio, (BMD) bone mineral density, and total porosity. n = 6. **E** Percentage of osteoclasts surface per bone surface (OCs/BS, %). n = 5. **F**, **G** BMDMs were incubated with Ti particles (0.1 mg/ml) and butyrate (1 mM) for 6 h after being primed with LPS (100 ng/ml) for 3 h, then cell lysates and supernatants were used for analysis of western blot. Cleaved Caspase-1 (P20) was detected from supernatant samples. NLRP3, Caspase-1 pro, IL-1β pro and Actin were detected in cell lysates (**F**). IL-1β were detected from supernatant samples (**G**). n = 3. **H** Representative immunohistochemical staining of NLRP3 and IL-1β. **I** Quantification of NLRP3 and IL-1β expression using integrated optical density/specimen area (IOD/Area). **J** Representative immunofluorescence staining of Caspase-1 in calvarial specimens. **K** Quantification of Caspase-1 using mean density (integrated density/specimen area) in calvarial specimens. n = 5. Results are expressed as mean ± SEM (One-way ANOVA[post hoc:SNK] **p < 0.01, ***p < 0.001)

As illustrated before, melatonin could enrich the fecal concentration of butyrate in Ti-particles exposed mice. However, whether butyrate was responsible for benefits of melatonin remains elusive. Therefore, we further investigated the response to butyrate supplementation in Ti-particles induced osteolysis model. We treated SPF mice with sodium butyrate in drinking water or control water (matched for pH and sodium content) for 2 weeks after the surgery procedure. Compared with Ti group, the bone erosion parameters (BV/TV, BMD, total porosity) were in a lower level after butyrate supplementation, indicating that butyrate supplementation could attenuate Ti-particles induced osteolysis (Fig. 4A–D). Meanwhile, H&E and TRAP staining also revealed less bone erosion, fewer osteoclasts activation and a lower ratio of OCs/BS after butyrate treatment (Fig. 4E, Additional file: Figure S4H, I). Thus, we could draw a conclusion that the anti-osteolysis effect of melatonin may rely on the enrichment of microbial metabolite butyrate.

Several studies had determined that the stimulation of NLRP3 inflammasome was strongly associated with the development of aseptic osteolysis [3–6]. To evaluate that the stimulation of NLRP3 inflammasome was indispensable in the development of osteolysis, caspase-1 inhibitor (Ac-YVAD-CMK) and NLRP3 inhibitor (MCC 950) were intraperitoneally injected into mice with calvarial osteolysis surgery to verify the impact of NLRP3 inflammasome on osteolysis in vivo*.* After two weeks, mice were sacrificed. Histomorphology and Micro-CT imaging analysis were employed to verify the involvement of NLRP3 inflammasome in the process of osteolysis. As discussed above, the result of Micro-CT 3D reconstruction revealed less bone erosion in mice received with either NLRP3 inhibitor (MCC 950) or Caspase-1 inhibitor (Ac-YVAD-CMK) (Additional file 1: Figure S4A). Further analysis bone erosion parameters also confirmed the protective effect of NLRP3 and caspase-1 inhibitors (Additional file 1: Figure S4B-D). H&E and TRAP staining also revealed the attenuation of osteolysis with less bone erosion and osteoclasts activation (Additional file 1: Figure S4E-G). These results confirmed that both caspase-1 and NLRP3 inhibitor could effectively block Ti-particles induced osteolysis. It is suggested that activation of NLRP3 inflammasome plays the essential part in Ti-particles induced osteolysis.

After determining the correlation between Ti-particles induced osteolysis and NLRP3 inflammasome activation, we intended to figure out whether the gut metabolite butyrate promoted by melatonin exerted the therapeutic effect against osteolysis through inhibiting the stimulation of NLRP3 inflammasome. Then We used BMDMs (bone marrow derived macrophages) for in vitro experiments. we detected the relative protein expression of NLRP3, Caspase-1 and IL-1β in butyrate treated BMDMs (LPS and Ti-particles primed). We found NLRP3 was inhibited in cell lysates (Fig. 4F). More importantly, cleaved caspase-1 (P20) and cleaved IL-1β in supernatants, parameters of NLRP3 inflammasome activation, were evidently suppressed after butyrate intervention (Fig. 4F, G). Next, to confirm the beneficial effects of butyrate in vivo, the expression of these proteins was detected in calvarial specimens. As shown in Fig. 4H–K, NLRP3, caspase-1, and IL-1β was remarkably inhibited in butyrate treated mice. The IOD/Area (integrated optical density/specimen area) value of NLRP3 and IL-1β was decreased by more than 66% in butyrate group (Fig. 4I). Additionally, the mean density (integrated density/specimen area) value of Caspase-1 decreased by almost 50% after two weeks of butyrate supplementation (Fig. 4K). All these results suggested that the anti-osteolysis effect of butyrate relied on suppression of the NLRP3 inflammasome.

### Butyrate alleviates osteolysis via its receptor GPR109A

**Fig. 5:**
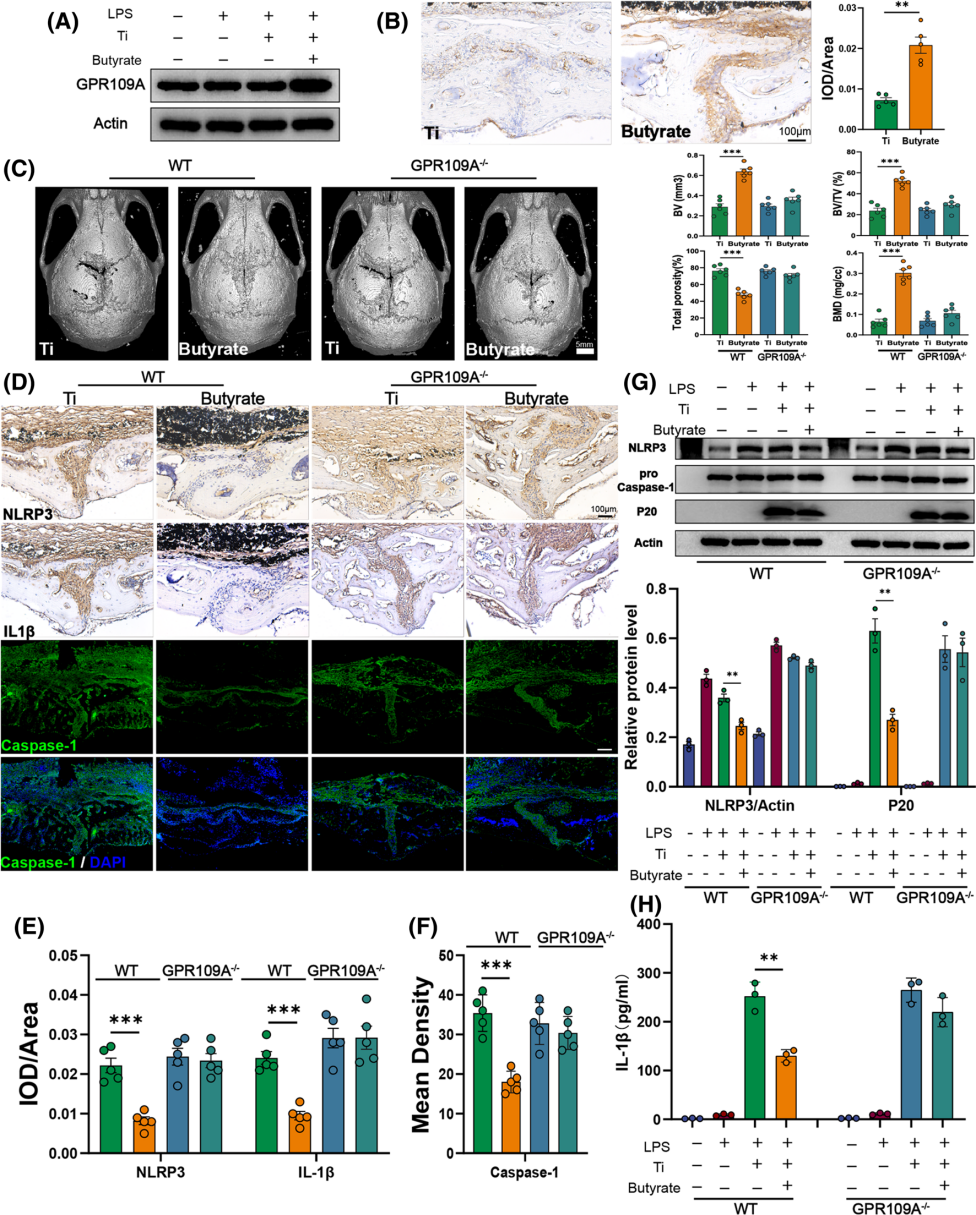
Butyrate exerted anti-osteolysis effects via its receptor GPR109A. **A** Western blot analysis of GPR109A in BMDMs. n = 3. **B** Representative immunohistochemical staining and quantification of GPR109A. n = 5. **C** Representative view of calvarium in each group via Micro-CT 3D reconstruction and quantification of bone erosion parameters (BV) bone volume, (BV/TV) bone volume to tissue volume ratio, (BMD) bone mineral density, and total porosity. n = 6. **D** Representative immunohistochemical staining of NLRP3, IL-1β and immunofluorescence staining of Caspase-1 in calvarial specimens. **E** Quantification of NLRP3 and IL-1β expression using integrated optical density/specimen area (IOD/Area). **F** Quantification of Caspase-1 using mean density (integrated density/specimen area) in calvarial specimens. n = 5. **G** BMDMs from wild type or GRP109A^−/−^ mice were incubated with Ti particles (0.1 mg/ml) and butyrate (1 mM) for 6 h after being primed with LPS (100 ng/ml) for 3 h, then cell lysates and supernatants were used for analysis of western blot. NLRP3, Caspase-1 pro, and Actin were detected in cell lysates. Cleaved Caspase-1 (P20) were detected from supernatant samples. n = 3. **H** The cytokine of IL-1β in supernatants was measured by ELISA. Results are expressed as mean ± SEM (One-way ANOVA[post hoc:SNK] **p < 0.01, ***p < 0.001)

As the main receptor, GPR109A was involved in homeostasis regulated by butyrate. We continued to determine whether anti-osteolysis of butyrate was mediated through the known receptor for butyrate, GPR109A. As shown in Fig. 5A, the expression of GPR109A was significantly up-regulated by butyrate in BMDMs. Furthermore, the immunohistochemical staining of GRP109A in calvarium also revealed an overexpression with a high value of IOD/Area after two weeks of butyrate intervention (Fig. 5B). Taken together, butyrate may exert anti-osteolysis effects by activating GPR109A.

To confirm the role of GPR109A in osteolysis, we compared the effects of butyrate intervention on osteolysis in GPR109A^−/−^ and wild type mice. We observed that the therapeutic effect of butyrate was greatly diminished in GPR109A^−/−^ mice. Compared with wild-type mice, GPR109A^−/−^ mice have almost the same bone erosion parameters (Fig. 5C). More importantly, butyrate could not block the activation of NLRP3 inflammasome in GPR109A^−/−^ mice (Fig. 5D). The value of IOD/Area and Mean Density in GPR109A^−/−^ mice did not show a different change after butyrate administration, which meant the expression of NLRP3, Caspase-1 and IL-1β were not affected by butyrate in GPR109A^−/−^ mice (Fig. 5E, F). And we also found the same phenomenon in BMDMs from GPR109A^−/−^ mice. Compared with BMDMs from wild type, the expression of NLRP3 in cell lysates and cleaved caspase-1 (P20) in supernatants showed no differences in BMDMs from GPR109A^−/−^ mice (Fig. 5G). Furthermore, the cytokine of IL-1β in cell supernatants was also not affected by butyrate (Fig. 5H). These results suggested an indispensable role for GPR109A in inhibiting NLRP3 inflammasome by butyrate, and thus attenuating osteolysis.

### The inhibitory effect of melatonin on inflammasome is mainly mediated by gut microbiota

**Fig. 6:**
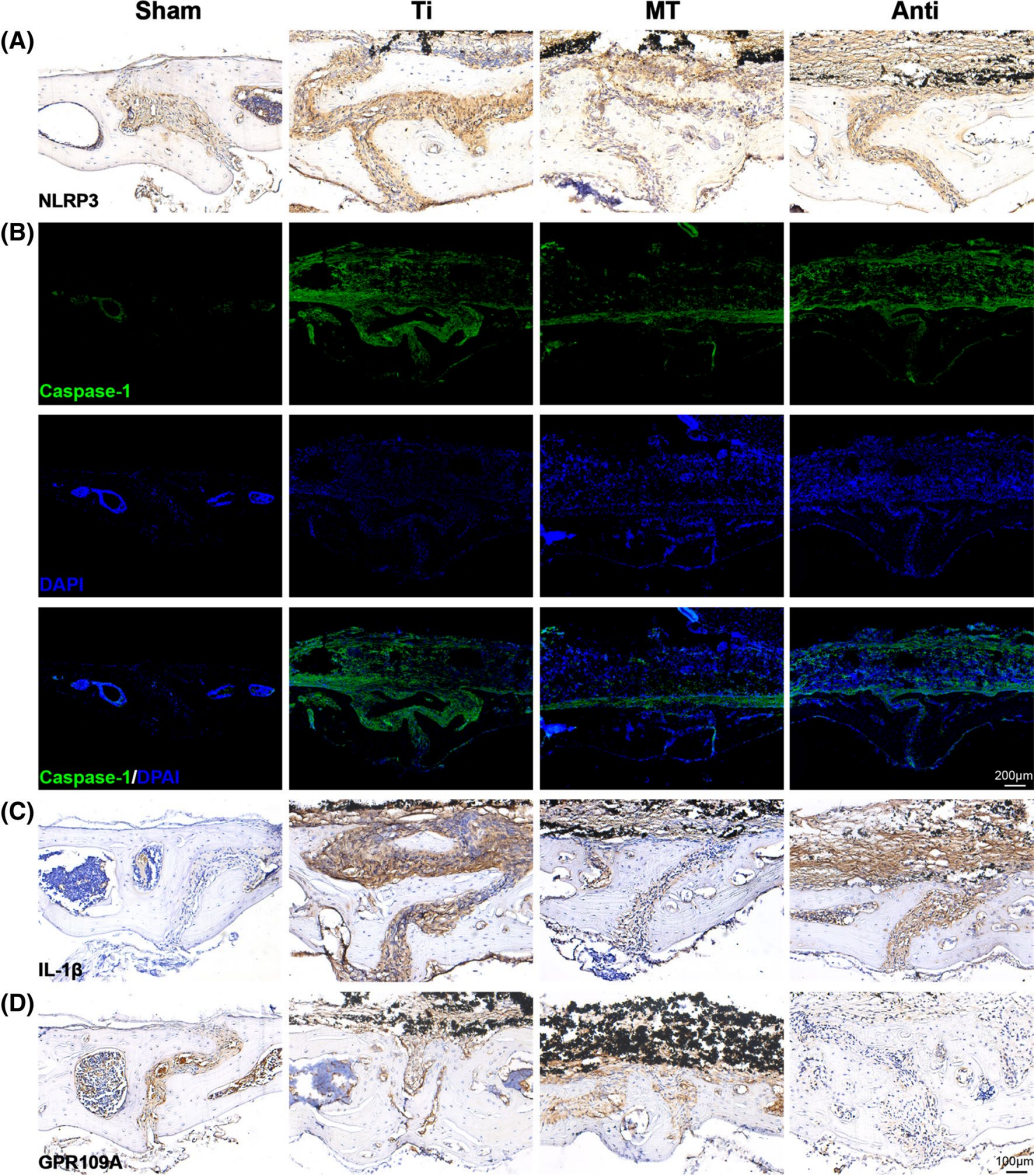
The ability of melatonin to inhibit NLRP3 inflammasome was evidently diminished by antibiotics. **A** Representative immunohistochemical staining of NLRP3 in calvarial specimens. **B** Representative immunofluorescence staining of Caspase-1 in calvarial specimens. **C** Representative immunohistochemical staining of IL-1β in calvarial specimens. **D** Representative immunohistochemical staining of GPR109A in calvarial specimens

Given that previous studies have shown melatonin has the ability to inhibit NLRP3 inflammasome, we compared the expression of inflammasome associated protein in sham group, Ti group, MT group and Anti group. Melatonin suppressed the expression of NLRP3, Caspase-1 and IL-1β, while antibiotics intervention with melatonin (Anti group) did not have an effective inhibitory in NLRP3 inflammasome activation (Fig. 6A–C, Additional file 1: Figure S5A-C). More importantly, the expression of GPR109A was up-regulated by melatonin administration compared with Ti group. As expected, the receptor of GPR109A was rarely expressed in Anti group with a lower concentration of butyrate (Fig. 6D, Additional file 1: Figure S5D). These data once again demonstrated that melatonin's ability of inhibiting inflammasome and alleviating osteolysis was mainly mediated by butyrate through gut microbiota.

## Discussion

Tremendous amounts of studies have confirmed that melatonin has a protective effect on the prevention and treatment of osteoporosis, arthritis, and other orthopedic diseases [42–45]. The beneficial effects of melatonin in Ti-particles induced aseptic inflammatory osteolysis has also been confirmed by our previous studies [25, 26]. In this study, we demonstrated that oral melatonin supplementation could alleviate osteolysis with a lower level of bone erosion parameters (BV/TV, BMD, total porosity), fewer osteoclasts activation and accompany with changes in gut microbiota.

Gut microbiota is closely correlated with systemic homeostasis, especially in the skeletal system [46–50]. Previous studies showed that melatonin could prevent obesity, improve lipid dysmetabolism, attenuate SD‐induced intestinal mucosa injury, alleviate weanling stress through regulating gut microbiota [21, 51–53]. However, whether melatonin can exert the anti-osteolysis benefits through gut microbiota is still unknown. Our results showed that melatonin administration could reshape the structure of gut microbiota, and reverse the dysbacteriosis triggered by osteolysis. Moreover, the results of antibiotic cocktail treatment and microbiota transplantation further confirmed that the benefits of melatonin were mediated by gut microbiota, which indicated an important role of gut microbiota in the development of osteolysis.

**Fig. 7:**
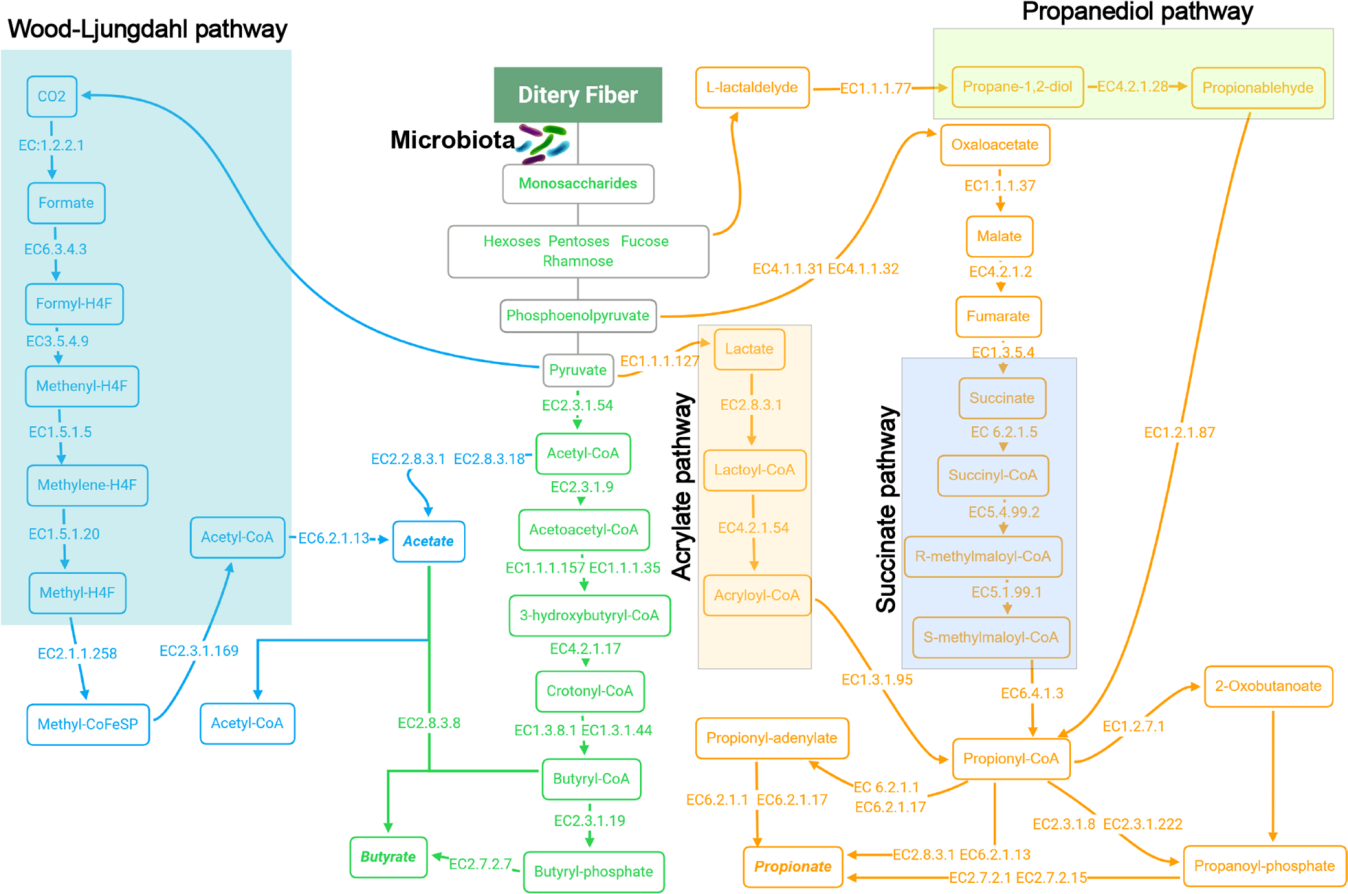
Metabolic pathways of three major SCFAs from carbohydrate fermentation. The gut microbiota synthetizes three major SCFAs, acetate, propionate, and butyrate through dietary fiber. Acetate is synthesized via the Wood-Ljungdahl pathway and pyruvate. Propionate can be produced from the propanediol, succinate, and acrylate pathway. Butyrate is formed through the molecule of acetyl-CoA

Analysis of 16S rRNA revealed melatonin administration could elevate the relative abundance of *Bifidobacterium, Bacteroides* and *Ruminococcus* genera, which are well known to be correlated with short chain fatty acids (SCFAs) production. As the metabolites of gut microbiota, SCFAs are the bridge between gut and bone metabolism as well as inflammatory diseases. Yin et al*.* demonstrated that melatonin increased the concentration of acetate in a high fat diet [21], while Lv et al*.* showed that three major SCFAs (acetate, propionate, and butyrate) all increased in DSS-induced depression rats [54]. However, our results showed that only butyrate was remarkably promoted by melatonin administration. Interestingly, no matter in which study, the increased SCFAs producing bacterium was different and associated with more than one kind of short chain fatty acid. But in the end, not all of the associated SCFAs would increase. For example, the relative abundance of *Bacteroides*, a genus of bacteria related to the synthesis of propionate, was increased in our study, but the concentration of propionate did not correspondently raise. More importantly, the genera of SCFAs producing bacteria enriched after transplantation was not completely consistent with the donors. Therefore, PICRUSt2, a metagenomic approach, was applied to predict the functional potential of a bacterial community on the basis of marker gene sequencing profiles. According to KEGG and metacyc databases, three major SCFAs are synthesized from dietary fibers with a lot of enzymes. Analysis of PICRUSt2 showed that melatonin promoted the expression of enzymes related to acetate synthesis by up-regulating acetyl-CoA mediated pyruvate pathway enzymes (EC:2.3.1.54, EC:2.8.3.1), rather than Wood-Ljungdahl pathway (EC:6.3.4.3, EC:1.5.1.5, EC:1.5.1.20, EC:2.1.1.258, EC:2.3.1.169). Consistent with the change of SCFA in fecal samples, the related enzymes of butyrate synthesis generally increased, while propionate related enzymes showed no tendency and some of them were even lower after melatonin administration (EC:5.4.99.2, EC:5.1.99.1, EC:6.2.1.1). Additionally, the same trends were also observed in microbiota transplantation. These results suggested the changes of SCFAs were not only affected by the shifting of bacterial community structure, but also related to the expression level of SCFA metabolic enzymes required for their synthesis (Fig. 7). It was the first time that the mechanism of how melatonin increased specific SCFAs has been clearly elucidated. More importantly, we found this ability could be transmitted by fecal transplantation. What particularly meaningful is that these findings provide another possible treatment for people who are not sensitive to dietary fiber fermentation or those who do not respond to probiotics replenishment.

Recently, several studies have confirmed that the activation of NLRP3 inflammasome in macrophages may be a dominant trigger in the development of aseptic osteolysis [3–6, 16]. Our results also confirmed that Ti-particles could highly promote the NLRP3 inflammasome activation in vitro and in vivo. Moreover, both NLRP3 inhibitor and caspase-1 inhibitor could effectively attenuate Ti-particles induced osteolysis in vivo, indicating that NLRP3 inflammasome may be a vital therapeutic target for prevention and treatment of prosthetic osteolysis. As reported in a large number of previous studies, butyrate could not only regulate inflammatory bowel disease [55], but also had an obvious therapeutic effect in other acute or chronic inflammatory diseases [56, 57]. For instance, Theiler et al*.* showed that butyrate ameliorated allergic airway inflammation [57]. The same anti-inflammatory effects of butyrate were also observed in caerulein-induced acute pancreatitis animal model [56]. Most of these protective effects on inflammation were attributed to the regulation on immune cells, especially in macrophages. As the dominant cells in osteolysis, macrophages released tremendous inflammation-related cytokines and then stimulated the formation and activation of osteoclasts [58]. As discussed above, the maturation of IL-1β, which was stimulated by the activation of caspase-1, was indispensable in the course of osteolysis [3–6, 16]. The effect of butyrate in osteoclast-related bone marrow derived macrophages was still unknown. In this study, we determined butyrate could evidently suppress the stimulation of NLRP3 inflammasome in BMDMs. Meanwhile, this beneficial effect was also observed in calvarial specimens via immunohistochemistry staining of IL-1β. Furthermore, we also confirmed that these benefits of butyrate on osteolysis were dependent on its receptor GPR109A.

After verifying the speculation of metabolite butyrate on osteolysis, we performed different immunostainings of calvarium collected from Sham, Ti, MT and Anti group. We found the suppression of melatonin in NLRP3 inflammasome related proteins were largely diminished when antibiotics were added into melatonin intervention. Combined with the results of antibiotics treatment, 16S rRNA, PICRUSt2, microbiota transplantation and immunostaining of inflammasome proteins in calvarium from Sham, Ti, MT and Anti groups, we may make a conclusion that the therapeutic effects of melatonin on osteolysis are largely mediated by regulating butyrate, a metabolite of gut microbiota.

## Conclusions

**Fig. 8:**
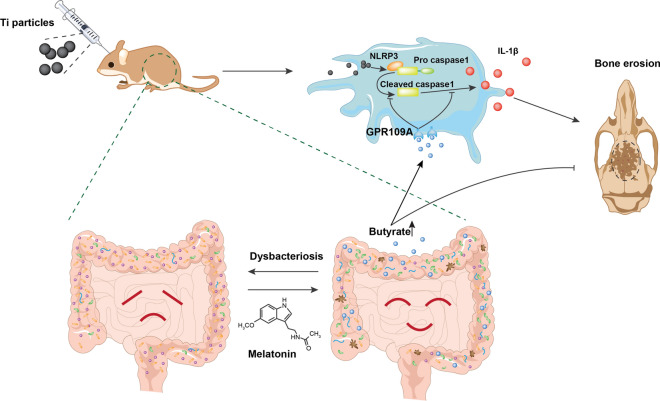
Melatonin alleviates Ti-particles induced osteolysis through the enrichment of gut microbiota metabolite butyrate. Melatonin reverses the dysbacteriosis triggered by inflammatory osteolysis and raises the relative abundance of SCFA producing bacterium. Enrichment of butyrate following the change of microbiota activates GPR109A and suppresses osteolysis via inhibiting NLRP3 inflammasome activation

In summary, our work highlighted the therapeutic effect of melatonin on Ti-particles induced osteolysis. Additionally, the mechanism that melatonin administration reversed the dysbacteriosis triggered by osteolysis, increased the relative abundance of SCFAs-producing microbes, enhanced the expression of butyrate synthesis enzymes, and enriched the fecal concentration of butyrate was fully uncovered for the first time. And the effective suppression of NLRP3 inflammasome activation mediated by butyrate enrichment was responsible for the therapeutic action of melatonin on osteolysis. Furthermore, the benefits of butyrate were dependent on its receptor GPR109A (Fig. 8). Thus, melatonin could be a qualified regulator of gut microbiota to exert anti-inflammatory osteolysis as well as the microbial metabolite butyrate.

## Methods

### Animal study

C57BL/6 J male mice (8–9 weeks) were purchased from Suzhou Healthytech Bio-pharmaceutical Co., Ltd. (Suzhou, China). All mice were maintained in a SPF (specific-pathogen-free) environment with free access to water and diets under the standard of 12 h-light and 12 h-dark cycle at 22 ± 1 °C. Mice in the following groups of Ti, MT, Anti, Ti-trans, MT-trans, Butyrate, MCC950, and Ac-YVAD-CMK were subjected to osteolysis surgery as previously described [28]. Briefly, titanium particles (Nanjing Emperor Nano Materials, China) were incubated under 180 °C for 8 h to remove endotoxins and transferred to ethanol solution for one day. The average size of the particles was 500 ± 20.2 nm (range 60–700 nm, median 380 nm). Then, titanium particles were resuspended in sterile PBS with the concentration of 500 mg/ml for subsequent studies. Each mouse’s calvarium received 40ul Ti-particles with a concentration of 500 mg/ml through a 10 mm midline sagittal incision in these groups and mice in Sham group received sham operation without treatment.

In antibiotic cocktail treatment study, forty mice (25.2 ± 0.3 g) were randomly divided into sham, Ti, MT, Anti groups. Mice in Anti group were treated with a mixture of antibiotics (0.5 g/L ampicillin, 1 g/L streptomycin, 0.5 g/L vancomycin, 1 g/L gentamicin) diluted in drinking water. Melatonin (Sangon Biotech, Shangshai, China) was diluted in drinking water with the concentration of 0.4 mg/ml. Each mouse drank average 6.53 ± 0.32 ml/day melatonin water. Thus, the approximate melatonin consumption in this study was about 109 mg/kg body weight/day, almost similar to previous study [21]. The consumption of melatonin in MT and Anti group was general equal. All antibiotics were bought from Sigma–Aldrich.

In fecal microbiota transplantation study, thirty mice were randomly assigned to MT-trans (fecal microbiota transplantation from mice with Ti-particles exposure and melatonin treatment) and Ti-trans (fecal microbiota transplantation from mice with Ti-particles exposure) groups. At first, every group received a same mixture of antibiotics in drinking water. After two weeks, water contained antibiotics was replaced with normal water. Meanwhile these mice were transplanted with fecal microbiota from donor mice in Ti and MT group respectively. Briefly, fecal samples were collected from Ti and MT groups. About 100 mg feces was re-suspended in 3.0 ml sterile PBS. Fecal samples were well mixed and then centrifuged. The microbiota supernatant from Ti and MT groups was transplanted into mice treated with antibiotics by gavaging for 15 days (0.2 ml/day). Then, mice were subjected to calvarial osteolysis surgery for two weeks and sacrificed for radiological and histological analysis after microbiota transplantation.

In butyrate treatment study, thirty mice were randomly assigned to sham, Ti, Butyrate groups. Mice in butyrate group received water with 150 mM sodium butyrate (Sigma–Aldrich St. Louis) as previously described [29]. Sham and Ti group supplied with matched for sodium content and pH water.

In the study of Caspase-1 and NLRP3 inhibitors intervention, forty mice were randomly divided to sham, Ti, Ac-YVAD-CMK (Santa Cruz) and MCC 950 (Santa Cruz) groups. Mice in Ac-YVAD-CMK group and MCC950 group were treated daily with Ac-YVAD-CMK (8 mg/kg body weight) or MCC 950 (10 mg/kg body weight) via intraperitoneal injection for two weeks after the surgery procedure according to previous studies [30, 31].

In the study of GPR109A receptor, twelve GPR109A^−/−^ mice and wild-type mice were randomly assigned to Ti and butyrate groups respectively. Mice in butyrate group received water with 150 mM sodium butyrate (Sigma–Aldrich St. Louis). Ti group supplied with matched for sodium content and pH water.

In the course of melatonin, antibiotic, and microbiota transplantation studies, feces from mice in every group were collected for further analysis.

### Micro-CT analysis

The detailed information is provided in the Supplementary data.

### 16S rRNA gene sequencing

The detailed information is provided in the Supplementary data.

### SCFAs (short-chain fatty acids)

Fecal samples were collected for quantification analysis of acetate, propionate, and butyrate by an Agilent 7890A gas chromatography (Agilent Technologies, USA) in Suzhou Bionovegene Co., Ltd., Jiangsu China.

### Cell culture and stimulation

The detailed information is provided in the Supplementary data.

### Enzyme-linked immunosorbent assay

IL-1β cell supernatants were measured by ELISA kit (MultiSciences) following the manufacturer’s instructions.

### Western blot analysis

The detailed information is provided in the Supplementary data.

### Histology, Immunohistochemistry and Immunofluorescence staining

The detailed information is provided in the Supplementary data.

### Statistical analysis

The statistical analysis was performed by a one-way analysis of variance (ANOVA) and post hoc Student–Newman–Keuls (SNK) test or an Unpaired t-tests within Sigmaplot 12.5 software (Systat Software, San Jose, CA, USA). Data are presented as mean ± SEM. P < 0.05 was considered statistically significant.

## Supplementary Information

**Additional file 1**: **Figure S1**. Melatonin induced change of gut microbiota in different phylogenetic levels, identified by LEfSe analysis. n=6. **Figure S2**. Fecal micobiota transplantation from melatonin treated mice could attenuate osteolysis. (A) H&E and (B) TRAP staining. (C-D) Relative abundance of acetate and propionate synthesis related enzymes from PICRUSt2 n = 6. Results are expressed as mean ± SEM (Unpaired t-tests * p < .05). (E) TRAP positive cells number and (F) percentage of osteoclasts surface per bone surface (OCs/BS, %). n = 5. Results are expressed as mean ± SEM (One-way ANOVA [post hoc:SNK] ** p < .01). **Figure S3**. Principle coordinate analysis (PCoA) plot between Ti-trans and MT-trans groups based on the Bray Curits distance. n = 6. **Figure S4**. Ti-particles induced osteolysis could be blocked by the treatment of either NLRP3 or caspase-1 inhibitor. (A) Representative view of calvarium from sham, Ti, MCC950, and Ac-YVAD-CMK groups via Micro-CT 3D reconstruction. (B-D) Quantification of bone erosion parameters (BV/TV) bone volume to tissue volume ratio, (BMD) bone mineral density, and total porosity. n=6. (E) Percentage of osteoclasts surface per bone surface (OCs/BS, %). n = 5.(F) H&E and (G) TRAP staining of calvarial slices from each group. (H) H&E and (I) TRAP staining of calvarial slices from Sham, Ti and Butyrate group. Results are expressed as mean ± SEM (One-way ANOVA[post hoc:SNK] *** p < .001). **Figure S5**. Quantification of NLRP3, Caspase-1, IL-1β and GPR109A staining. (A) Quantification of NLRP3 immunohistochemical staining using integrated optical density/specimen area (IOD/Area). n=5. (B) Quantification of Caspase-1 immunofluorescence staining using mean density (integrated density/specimen area). n=5. (C) Quantification of IL-1β immunohistochemical staining using integrated optical density/specimen area (IOD/Area). n=5. (D) Quantification of GPR109A immunohistochemical staining. n=5. Results are expressed as mean ± SEM (One-way ANOVA [post hoc:SNK] ** p < .01, *** p < .001).

## Data Availability

The datasets generated and/or analysed during the current study are not publicly available but are available from the corresponding author on reasonable request.
