# Supplementary material for: Melatonin alleviates titanium nanoparticles induced osteolysis via activation of butyrate/GPR109A signaling pathway
Source: J Nanobiotechnology. 2021 Jun 6;19:170. doi: 10.1186/s12951-021-00915-3 (PMC8182936; doi:10.1186/s12951-021-00915-3)
Supplement: Supplementary file 1 — Additional file 1: Figure S1. Melatonin induced change of gut microbiota in different phylogenetic levels, identified by LEfSe analysis. n=6. Figure S2. Fecal micobiota transplantation from melatonin treated mice could attenuate osteolysis. (A) H&E and (B) TRAP staining. (C-D) Relative abundance of acetate and propionate synthesis related enzymes from PICRUSt2 n = 6. Results are expressed as mean ± SEM (Unpaired t-tests * p < .05). (E) TRAP positive cells number and (F) percentage of osteoclasts surface per bone surface (OCs/BS, %). n = 5. Results are expressed as mean ± SEM (One-way ANOVA [post hoc:SNK] ** p < .01). Figure S3. Principle coordinate analysis (PCoA) plot between Ti-trans and MT-trans groups based on the Bray Curits distance. n = 6. Figure S4. Ti-particles induced osteolysis could be blocked by the treatment of either NLRP3 or caspase-1 inhibitor. (A) Representative view of calvarium from sham, Ti, MCC950, and Ac-YVAD-CMK groups via Micro-CT 3D reconstruction. (B-D) Quantification of bone erosion parameters (BV/TV) bone volume to tissue volume ratio, (BMD) bone mineral density, and total porosity. n=6. (E) Percentage of osteoclasts surface per bone surface (OCs/BS, %). n = 5.(F) H&E and (G) TRAP staining of calvarial slices from each group. (H) H&E and (I) TRAP staining of calvarial slices from Sham, Ti and Butyrate group. Results are expressed as mean ± SEM (One-way ANOVA[post hoc:SNK] *** p < .001). Figure S5. Quantification of NLRP3, Caspase-1, IL-1β and GPR109A staining. (A) Quantification of NLRP3 immunohistochemical staining using integrated optical density/specimen area (IOD/Area). n=5. (B) Quantification of Caspase-1 immunofluorescence staining using mean density (integrated density/specimen area). n=5. (C) Quantification of IL-1β immunohistochemical staining using integrated optical density/specimen area (IOD/Area). n=5. (D) Quantification of GPR109A immunohistochemical staining. n=5. Results are expressed as mean ± SEM (One-way ANOVA [post ho [file 12951_2021_915_MOESM1_ESM.docx]

**Supporting information**

**Melatonin alleviates titanium nanoparticles induced osteolysis via activation of butyrate/GPR109A signaling pathway**

Running title: melatonin alleviates osteolysis via butyrate

Yanglin Wu^1†^, Fan He^2†^, Chenhui Zhang^1^, Qin Zhang^1^, Xinlin Su^1^, Xu Zhu^1^, Ang Liu^1^, Weidong Shi^1^, Weifeng Lin^4^, Zhongqin Jin^3*^, Huilin Yang^1*^, Jun Lin^1*^

^1^ Department of Orthopaedics, The First Affiliated Hospital of Soochow University, Soochow University, Suzhou 215006, China

^2^ Orthopaedic Institute, Medical College, Soochow University, Suzhou 215007, China

^3^ Department of digestive, Children's Hospital Affiliated to Soochow University, Suzhou, China

^4^ Department of Materials and Interfaces, Weizmann Institute of Science, Rehovot 76100, Israel.

^†^ These authors contributed equally.

**Corresponding Authors:**

**^*^** Jun Lin, M.D., Ph.D., Department of Orthopaedics, The First Affiliated Hospital of Soochow University, No. 188 Shizi Street, Suzhou 215006, Jiangsu, China. Telephone: +86-512-67781420; Fax: +86-512-67781165; Email: linjun@suda.edu.cn

**^*^** Huilin Yang, M.D., Ph.D., Department of Orthopaedics, The First Affiliated Hospital of Soochow University, No. 188 Shizi Street, Suzhou 215006, Jiangsu, China. Telephone: +86-512-67781420; Fax: +86-512-67781165; Email: suzhouspine@163.com

**^*^** Zhongqin Jin, M.D., Department of digestive, Children's Hospital Affiliated to Soochow University, Suzhou, China, Email: sunyu0628@126.com

1. Experimental procedures
   1. Micro-CT analysis

calvarium collected from every group were subjected to Micro-CT (micro-computed tomography) scanning (SkyScan 1176, Aartselaar, Belgium). The parameters of X-Ray were set at a current of 500 µA with the voltage of 50 kV and the scanning per layer was 9 µm. Region of interest (ROI) of a 3 mm diameter round on every mouse calvarium was selected for quantification of bone erosion parameters (BV/TV, %) bone volume to tissue volume ratio, (BMD, mg/cc) bone mineral density, and total porosity (%).

- 1. 16S rRNA gene sequencing

CTAB/SDS method was used to extract total genome DNA from fecal samples for the amplification of 16S rRNA V3-V4 region by specific primer with barcode. PCR reactions were performed with 10 ng template DNA; 0.2 µM forward and reverse primers, and 15 µl PCR Master Mix (New England Biolabs). 1X loading buffer with the same volume of PCR products was added and the mixture was purified with Qiagen gel extraction kit (Qiagen, Germany). The sequencing library was prepared using TruSeq DNA PCR-Free sample preparation kit (Illumina, USA). An Illumina NovaSeq platform was used to sequence the library (Suzhou Bionovegene Co., Ltd., Jiangsu China).

- 1. Cell culture and stimulation

Bilateral femurs and tibiae of mice were isolated to obtain bone marrow-derived macrophages (BMDMs). Both ends of femurs and tibiae were cut to flush the bone marrow. The liquid collected from bone marrow was subjected to red blood cell lysis. Then bone marrow cells were resuspended in DMEM supplemented with 10% FBS and 50 ng/ml M-CSF (R&D Systems, USA) and then placed into six-well plate at 2–4 × 10^6^ cells/ml. The cells were subjected to further studies after five to seven days culture. To activate the inflammasome in BMDMs, the cells were stimulated with Ti particles in a concentration of 0.1mg/ml for 6h after incubation with 100 ng/ml LPS for 3 h. The concentration of butyrate intervention was 1mM according to previous study ^24^.

- 1. Western blot assay

Cells were lysed in RIPA lysis buffer (Beyotime, China) containing proteinase and phosphatase inhibitors and a BCA assay (Beyotime, China) was used to measure the concentration of protein. The supernatant proteins were precipitated. Protein of lytic samples were transferred to PVDF membranes (Beyotime, China) after separating by SDS-PAGE (Beyotime, China). Membranes were incubated with the anti-NLRP3 (Abcam, Camb, UK), anti-procaspase1, anti-cleaved caspase-1, anti-IL 1β (Cell Signaling Technology, MA, USA)， anti-GPR109A (Santa Cruz Biotechnology), anti-Actin (Beyotime, China) at 4°C overnight following the block with quick block buffer (beyotime, China) for thirty minutes. Then membranes were subjected to the secondary antibody for one hour in room temperature after washing three times with TBS-Tween. The result were visualized via chemiluminescent peroxidase substrate (Sigma-Aldrich).

- 1. Histology, Immunohistochemistry and Immunofluorescence staining

The mouse calvarium were embedded with paraffin. The specimen was cut to slices every 6 µm. H&E (hematoxylin and eosin) staining was guided by manufacturer’s instruction. TRAP staining was conducted by a tartrate-resistant acid phosphatase kit (Sigma-Aldrich) to detect osteoclasts. Image J was applied to quantify osteoclasts surface per bone surface ratio (OCs/BS, %).

For immunohistochemistry, 6µm slices of calvarial sections were cut and preform heat-induced antigen retrieval. Then the slices of calvarial section were incubation with IL-1β (rabbit, Cell Signaling Technology) antibody. Following incubation with primary antibody at 4°C overnight, the slices were subjected to the second antibody for 1 h at room temperature and stained for 2-6 minutes within 100μl DAB. Then took photos with a microscope.

For immunofluorescence, calvarial sections were incubated with NLRP3 (rabbit, Abcam) and Caspase-1(mouse, Santa Cruz) at 4°C overnight. Following washing with PBS for three time, calvarial slices were incubated with goat anti-Rabbit IgG and gota anti-Mouse IgG secondary antibody (Abcom Camb, UK) and DAPI (Beyotime, China) for 90 min and 10 min, respectively.

**
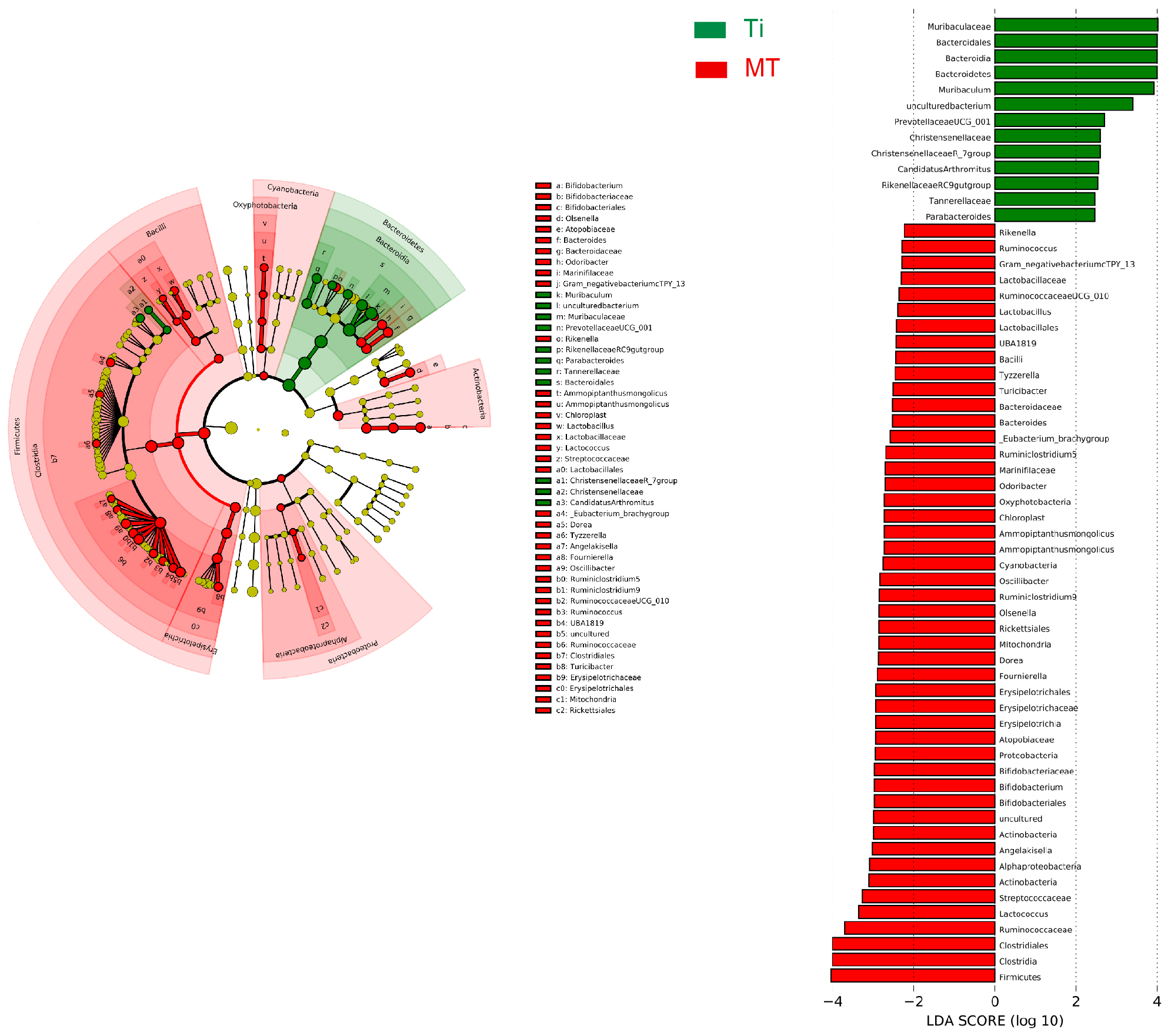
**

**Supporting information Figure S1.** Melatonin induced change of gut microbiota in different phylogenetic levels,identified by LEfSe analysis. n=6.


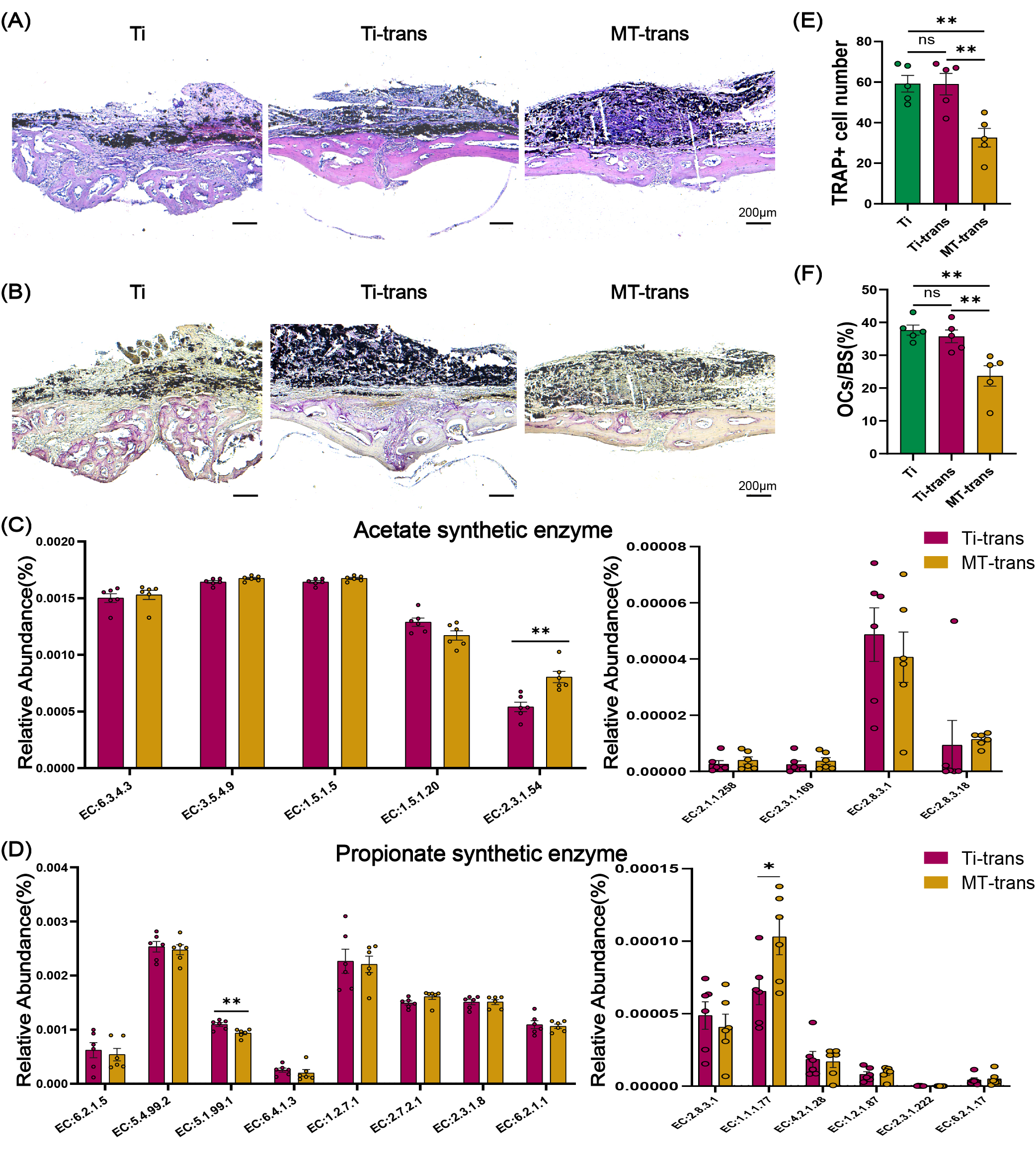


**Supporting information Figure S2.** Fecal micobiota transplantation from melatonin treated mice could attenuate osteolysis. **(A)** H&E and **(B)** TRAP staining. **(C-D)** Relative abundance of acetate and propionate synthesis related enzymes from PICRUSt2 n = 6. Results are expressed as mean ± SEM (Unpaired t-tests * p < .05). **(E)** TRAP positive cells number and **(F)** percentage of osteoclasts surface per bone surface (OCs/BS, %). n = 5. Results are expressed as mean ± SEM (One-way ANOVA[post hoc:SNK] ** p < .01).


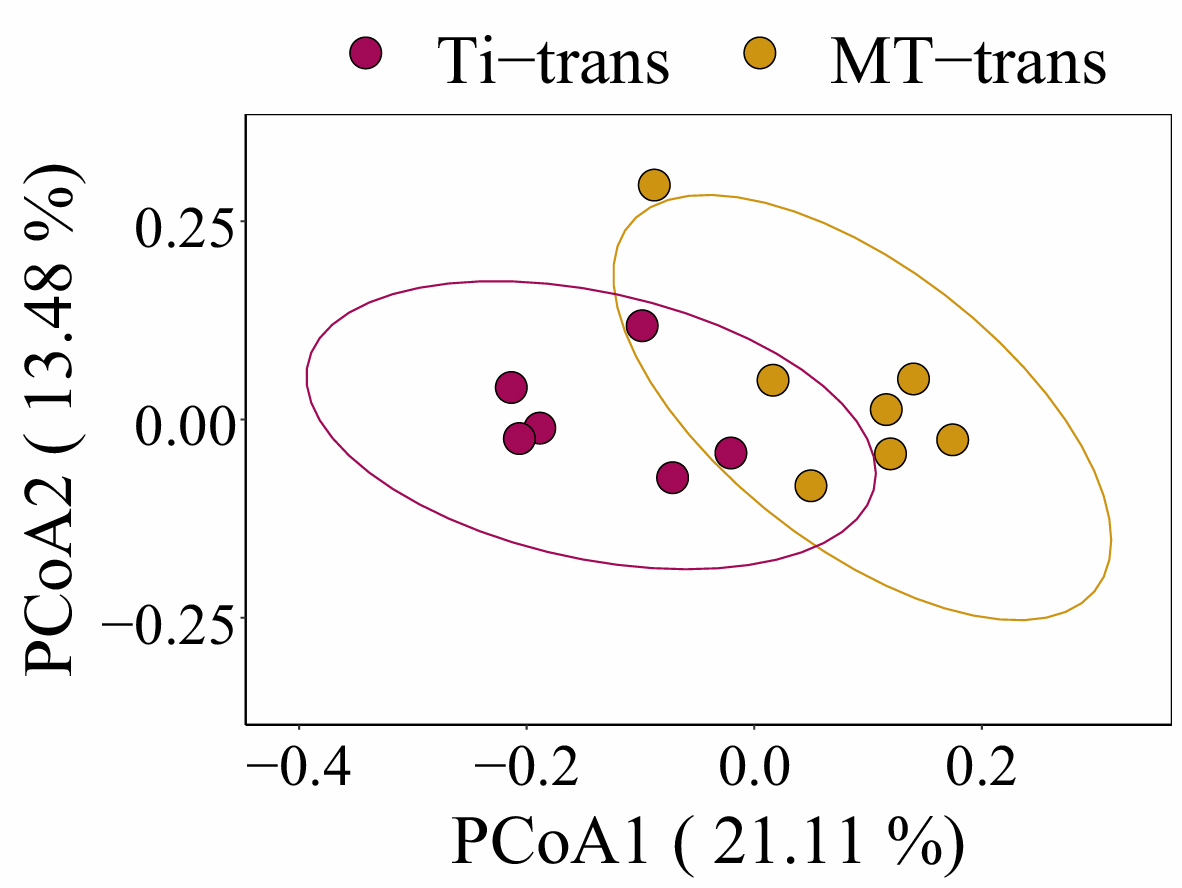


**Supporting information Figure S3.** Principle coordinate analysis (PCoA) plot between Ti-trans and MT-trans groups based on the Bray Curits distance. n = 6.


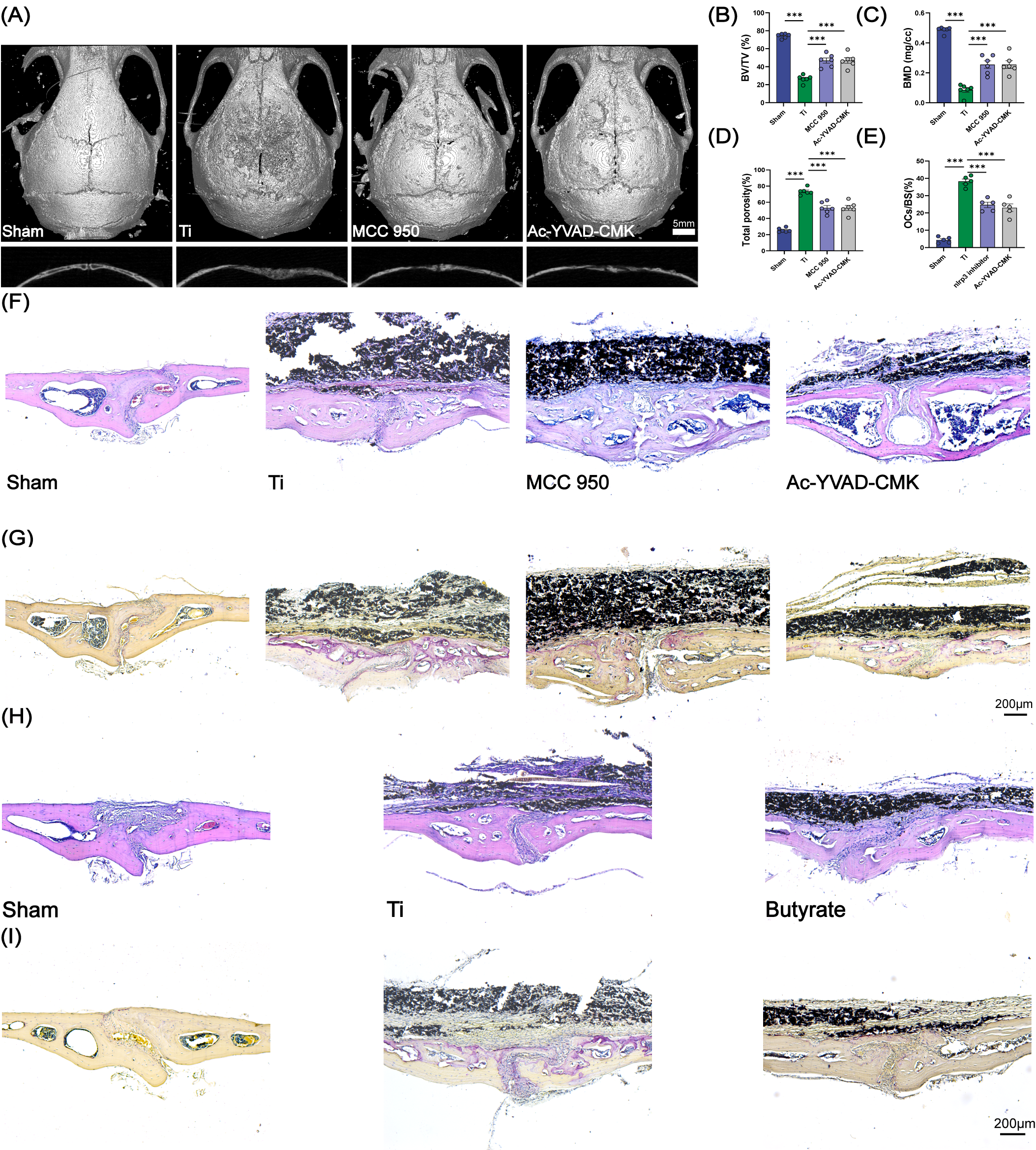


**Supporting information Figure S4** Ti-particles induced osteolysis could be blocked by the treatment of either NLRP3 or caspase-1 inhibitor. **(A)** Representative view of calvarium from sham, Ti, MCC950, and Ac-YVAD-CMK groups via Micro-CT 3D reconstruction. **(B-D)** Quantification of bone erosion parameters (BV/TV) bone volume to tissue volume ratio, (BMD) bone mineral density, and total porosity. n=6. **(E)** Percentage of osteoclasts surface per bone surface (OCs/BS, %). n = 5.**(F)** H&E and **(G)** TRAP staining of calvarial slices from each group. **(H)** H&E and **(I)** TRAP staining of calvarial slices from Sham, Ti and Butyrate group. Results are expressed as mean ± SEM (One-way ANOVA[post hoc:SNK] *** p < .001).


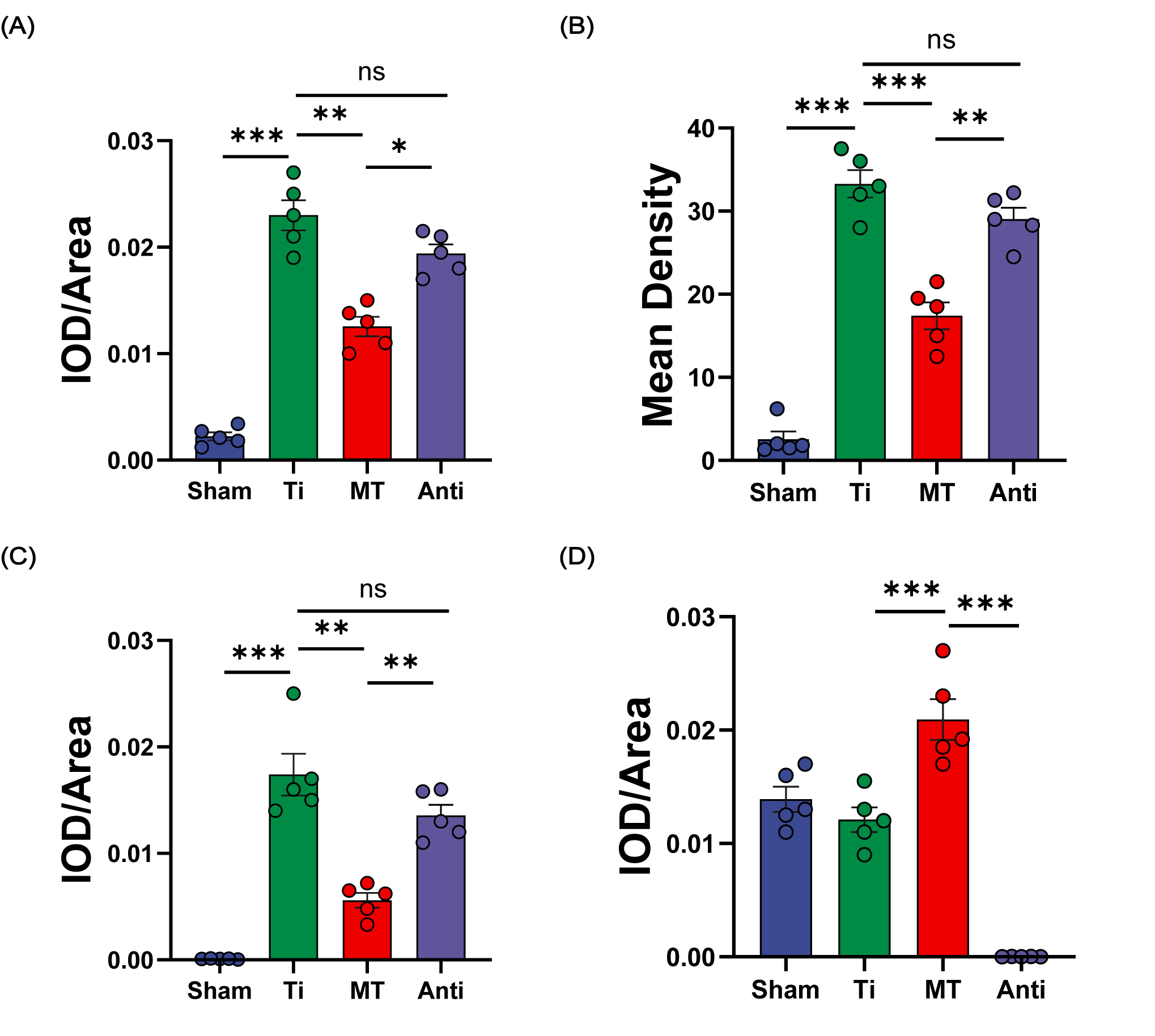


**Supporting information Figure S5** Quantification of NLRP3, Caspase-1, IL-1β and GPR109A staining. **(A)** Quantification of NLRP3 immunohistochemical staining using integrated optical density/specimen area (IOD/Area). n=5. **(B)** Quantification of Caspase-1 immunofluorescence staining using mean density (integrated density/specimen area). n=5. **(C)** Quantification of IL-1β immunohistochemical staining using integrated optical density/specimen area (IOD/Area). n=5. **(D)** Quantification of GPR109A immunohistochemical staining. n=5. Results are expressed as mean ± SEM (One-way ANOVA[post hoc:SNK] ** p < .01, *** p < .001).
